# Persistent Penile Erection Following Spinal or General Anesthesia During Pediatric Hypospadias Repair: A Case Report

**DOI:** 10.7759/cureus.72445

**Published:** 2024-10-26

**Authors:** Nimisha Cherunghattil, Jitendra V Kalbande, Pradeep Khobragade, Jakkireddy Sravani, Nitinkumar Borkar

**Affiliations:** 1 Anesthesiology, All India Institute of Medical Sciences, Raipur, Raipur, IND; 2 Anesthesiology and Critical Care, All India Institute of Medical Sciences, Raipur, Raipur, IND; 3 Anesthesiology, Critical Care, and Pain Medicine, All India Institute of Medical Sciences, Raipur, Raipur, IND; 4 Pediatric Surgery, All India Institute of Medical Sciences, Raipur, Raipur, IND

**Keywords:** dorsal penile block, general anaesthesia, gycopyrolate, hypospadiasis, intracavernous phenylephrine, ketamine, persistent penile erection, priapism, spinal anaesthesia

## Abstract

Penile erection (PE) during anesthesia is a rare yet challenging complication, particularly in pediatric patients undergoing urological surgeries such as hypospadias repair. This case report presents the management of a persistent PE in a 13-year-old male during stage II hypospadias repair under spinal anesthesia, which was subsequently switched to general anesthesia due to the failure of initial interventions. Despite attempts to resolve the erection using intravenous glycopyrrolate, ketamine, cold compresses, blood aspiration, muscle relaxation under general anesthesia, and dorsal penile nerve block, detumescence was not achieved. Finally, the administration of an intracavernous injection of phenylephrine resulted in complete detumescence, allowing the surgery to proceed without further complications. Therefore, this case highlights the early and firsthand use of intracavernous phenylephrine as a safe, simple, and effective method for managing persistent PE in pediatric surgical patients. It also underscores the importance of being prepared and adaptable when handling rare intraoperative complications during pediatric surgeries.

## Introduction

Hypospadias is a common congenital anomaly characterized by the abnormal placement of the urethral opening on the ventral aspect of the penis, rather than at the tip. In small pediatric patients with hypospadias, presenting early in life, repair is typically performed under regional anesthesia (such as caudal or dorsal penile nerve block) following deep sedation or general anesthesia. For older children, spinal anesthesia is often used for hypospadias repair due to its effectiveness and relatively low complication rate. However, penile erection (PE) following spinal anesthesia during hypospadias repair is an exceedingly rare occurrence [1]. Intraoperative PE, though uncommon, can manifest during regional or general anesthesia, leading to partial or complete erection during penile surgeries [2]. This condition poses significant challenges for the surgical team, as continuing with the procedure under such circumstances increases the risk of complications, including excessive bleeding, urethral trauma, and surgical closure. Consequently, surgery may need to be delayed or postponed to prevent these adverse outcomes [3,4].

Pediatric surgeons and anesthesiologists should be aware of this rare event and collaborate closely to manage and reverse it effectively. While most documented cases of intraoperative priapism have been reported during adult urological procedures, its occurrence in pediatric patients, particularly during hypospadias repair, is rarely mentioned in the literature [1,5]. This case report presents the successful management strategies used to address resistant PE in pediatric patients undergoing hypospadias repair under spinal followed by general anesthesia.

## Case presentation

A 13-year-old male patient, weighing 40 kg and standing 152 cm tall, with an American Society of Anesthesiologists (ASA) Physical Status I classification, was scheduled for a stage II hypospadias repair. He had previously undergone a stage I hypospadias repair six months earlier for penoscrotal hypospadias with severe chordee under spinal anesthesia, which was uneventful. All his preoperative investigations and vitals were within normal limits. The child was kept nil per oral preoperatively as per ASA guidelines, was counseled about regional anesthesia and the surgery, and consent was obtained from the parents.

On the day of surgery, the child was again counseled about spinal anesthesia, moderately sedated in the pre-operative room with intravenous midazolam (0.05 mg/kg), and shifted to the operation theater. ASA standard monitors were attached, and baseline parameters were recorded. The child was then placed in a lateral position and under strict aseptic precautions; spinal anesthesia was administered using 2 ml of 0.5% hyperbaric bupivacaine with 20 micrograms of fentanyl through a 26-gauge Quincke’s needle at the L4-L5 interspace. The sensory block was achieved up to the T8 level before the start of surgery.

**Figure 1:**
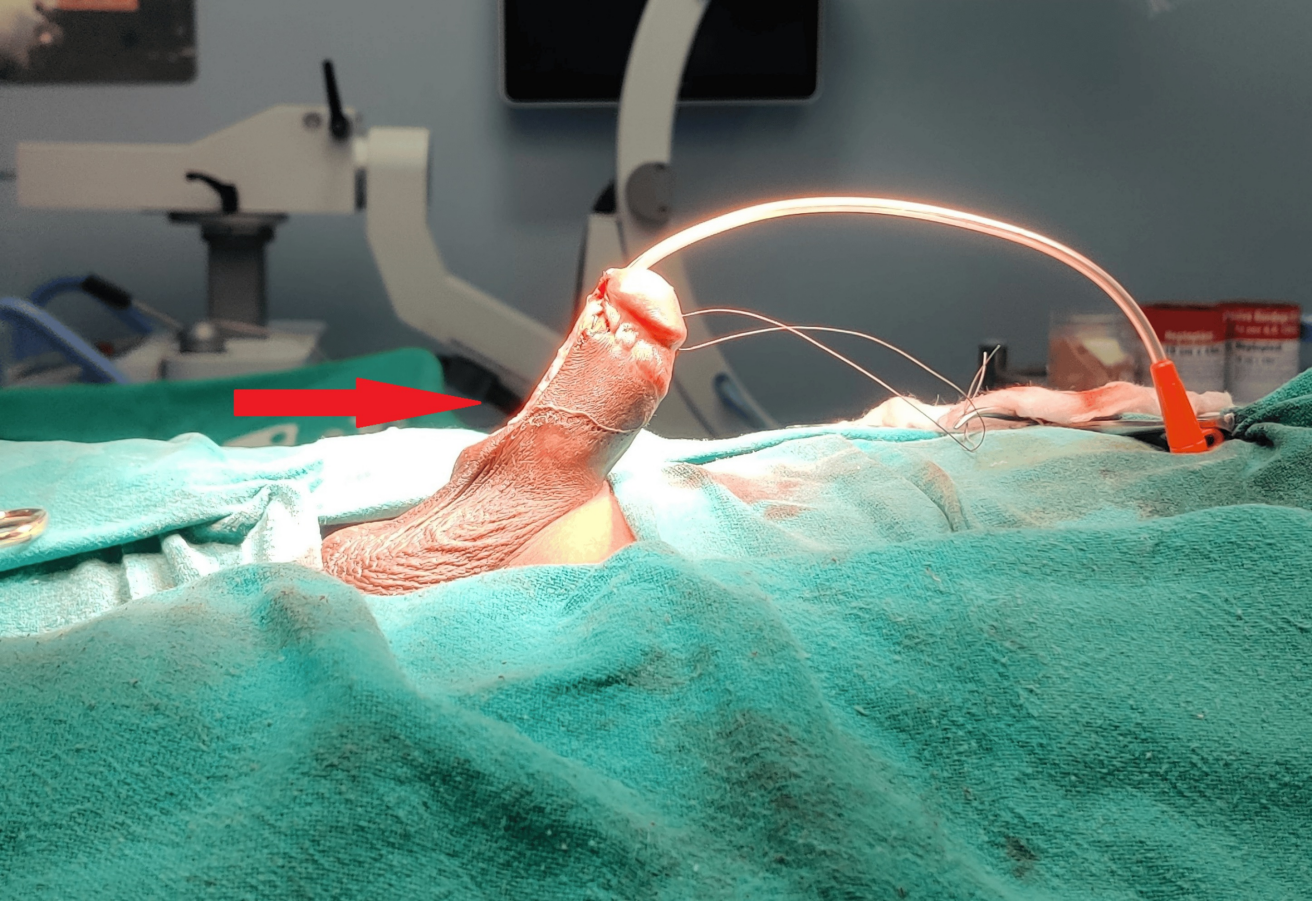
Red arrow indicating persistent penile erection even after treatment with injectable glycopyrrolate and ketamine.

After the painting and draping, the surgeon observed a PE 15 minutes after administering spinal anesthesia. Although the surgery commenced, the surgeon encountered difficulties due to excessive bleeding in the operative field. The persistent PE was promptly communicated to the anesthesia team by the surgeon. Thirty minutes after administering spinal anesthesia, 0.2 mg of IV glycopyrrolate was administered, and after waiting for 5 minutes, the erection persisted. Intraoperatively, the child was asked about any sensation of touch at the operative site, feelings of fear, stress, anxiety, excitement, emotionality, or dreams, all of which were denied. The child remained calm and was lying comfortably. Subsequently, ketamine was given in doses of 20 mg, 20 mg, and 10 mg at 3-minute intervals, totaling 50 mg for deep sedation (Richmond Agitation and Sedation Scale (RASS) score, -4), but the erection still did not resolve (Figure 1).

Oxygen supplementation at a rate of 4 L/min was given using a face mask, and ETCO2 was monitored during deep sedation. Meanwhile, the surgeon applied cold compresses and attempted to aspirate 5 ml of blood from the base of the corpus cavernosum with a syringe. However, the erection persisted, preventing the surgeon from proceeding with the planned surgical steps, including glansplasty and penile skin closure. Consequently, one hour after the spinal anesthesia, general anesthesia was induced using sevoflurane. Muscle relaxation was achieved with the administration of 15 mg of atracurium, and the patient's airway was secured with a size 2.5 I-Gel. Anesthesia was maintained with sevoflurane, oxygen, and air. Despite these interventions, full detumescence was not achieved, necessitating a therapeutic approach.

**Figure 2:**
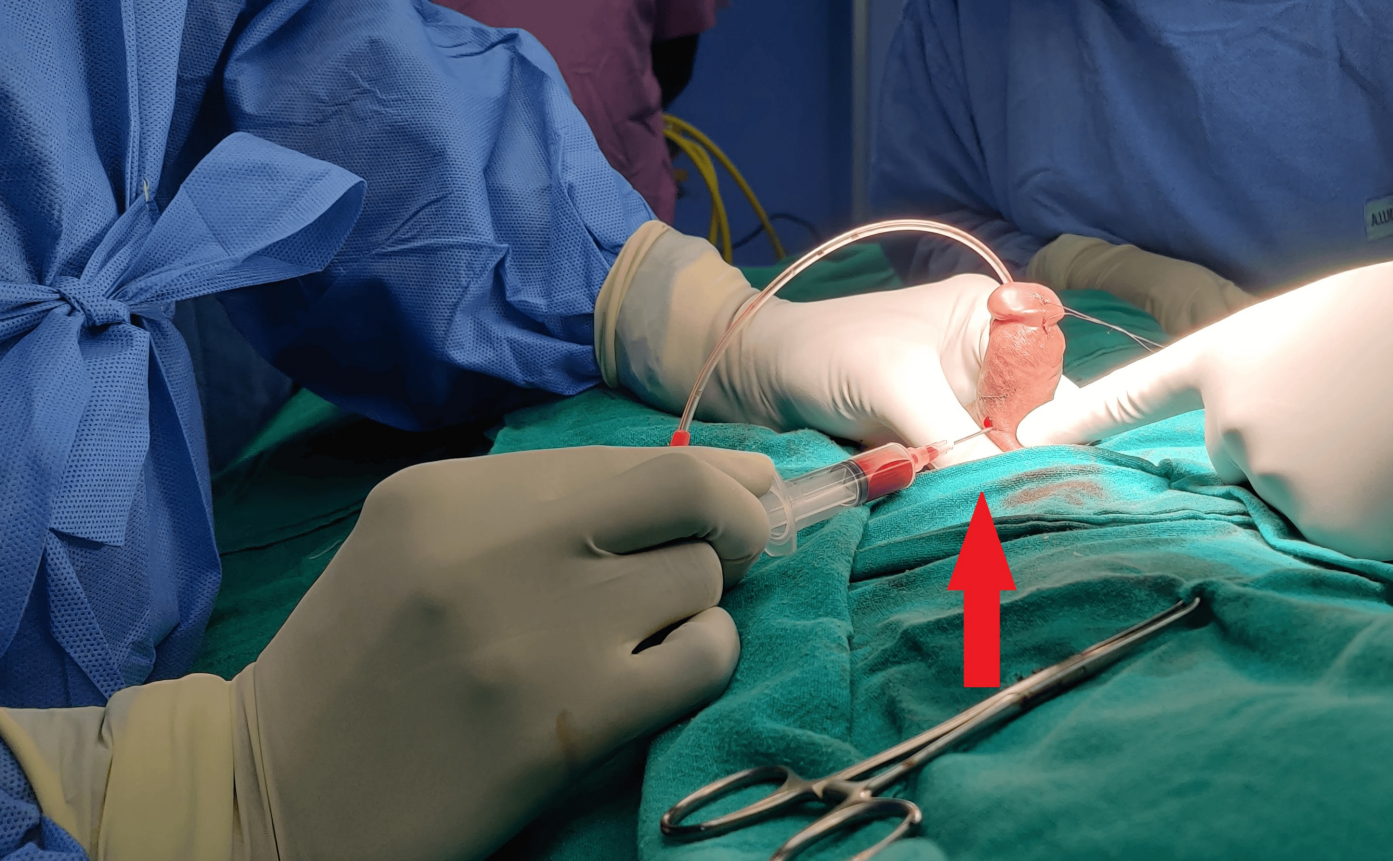
Red arrow indicating intracorporeal injection of phenylephrine for persistent penile erection.

**Figure 3:**
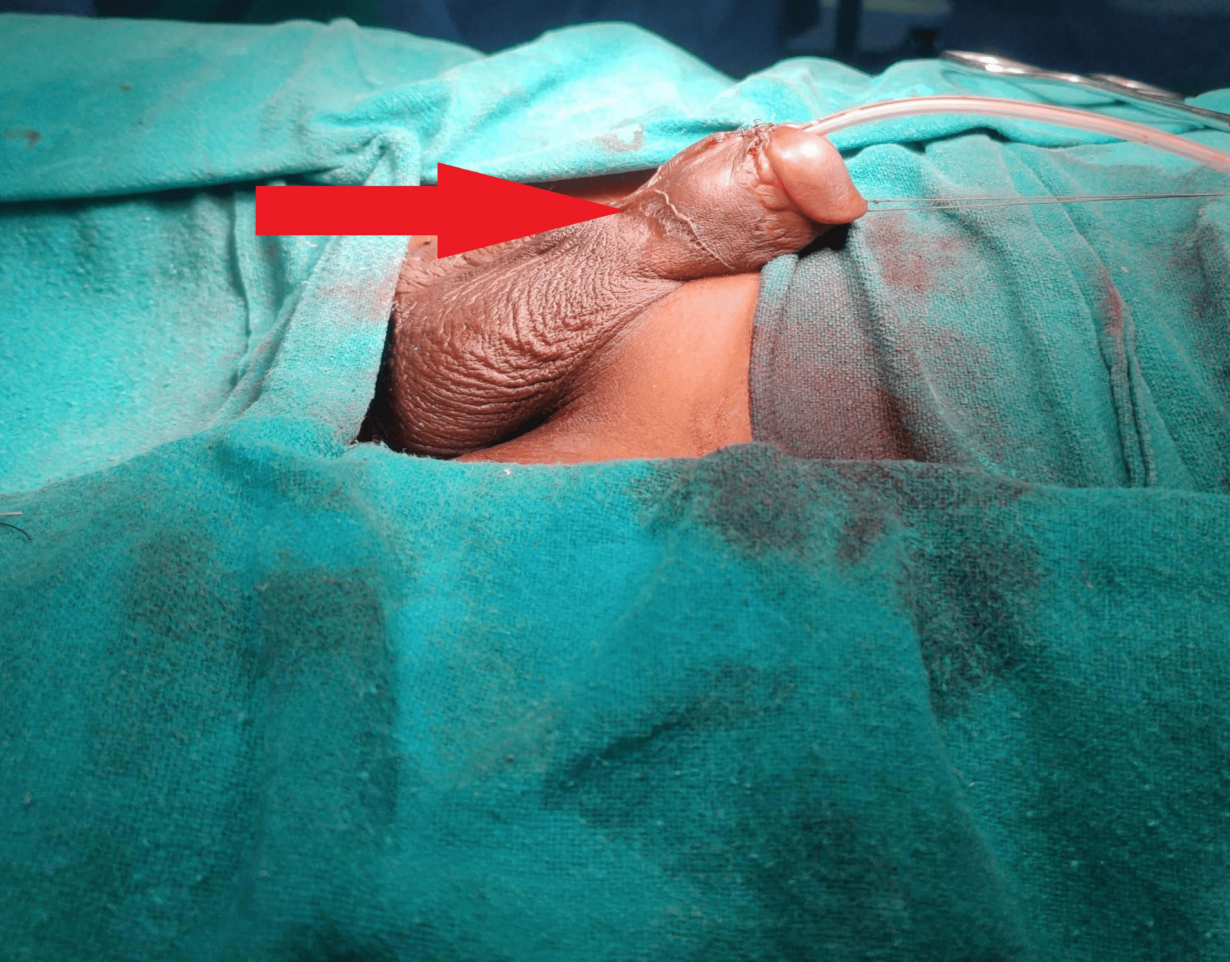
Red arrow showing penile detumescence and completion of surgery following intracorporeal injection of phenylephrine.

Following the initial 1.5 hours of the surgical procedure, the surgeon administered a dorsal penile nerve block with 2.5 ml of 2% lidocaine bilaterally. This intervention led to some degree of penile detumescence, but the surgical closure remained challenging. Subsequently, an intracorporeal injection of 50 micrograms of phenylephrine was administered, which resulted in complete detumescence within the next few minutes (Figures 2-3).

This allowed the surgery to be completed in 2.5 hours without further complications. After the procedure, the patient was reversed with injections of neostigmine (2 mg) and glycopyrrolate (0.2 mg) and was extubated once spontaneous respiration and airway reflexes were restored (Table 1).

**Table 1:** Sequence of treatment given for persistent penile erection.

	Time Points	Treatment given for persistent penile erection
1	0 min	Spinal anesthesia administered
2	15 min	Penile erection observed
3	30 min	Inj. Glycopyrrolate 0.2 mg administered
4	45 min	Inj. Ketamine 50 mg, cold compression, and blood aspiration from corpus cavernosum
5	60 min	General anesthesia induced with sevoflurane and Inj. Atracurium
6	90 min	Dorsal penile nerve block performed
7	105min	Intracavernous injection of phenylephrine 50 μg administered
8	110 min	Complete detumescence achieved
9	150 min	Surgery successfully completed
10	180 min	Patient extubated

The patient's vital signs remained stable throughout the perioperative period. Intravenous paracetamol at 15 mg/kg, administered three times a day, was given for postoperative analgesia. In the postoperative period, the patient's vitals, pain, and surgical site were closely monitored for oozing and recurrence of swelling over the subsequent 24 hours. The patient was then discharged home on postoperative day 5 and was reported to be doing well without any urinary symptoms at the one-month follow-up visit.

## Discussion

Hypospadias repair is a routine urological procedure in pediatric surgery, often conducted under spinal anesthesia due to its efficacy and relatively low complication rates. Priapism, or persistent PE unrelated to sexual stimulation, is defined as a condition that, if left untreated for more than four hours, can lead to edema, abrasion, tissue desiccation, and eventual penile necrosis [1,2]. Erection may occur irrespective of the type of anesthetic method employed. The reported incidence of intraoperative PE is 0.34% for general anesthesia, 0.11% for spinal anesthesia, and 1.72% for epidural anesthesia, with a higher prevalence in young males [2,3]. Penile engorgement poses significant challenges during urological procedures, potentially leading to delays, increased risks of complications such as bleeding, and placing the surgical sutures under tension, which results in improper healing and subsequent urethral stricture or fistula formation, or even cancellation of the scheduled operation. In our case, this complication proved challenging, as it obstructed the surgical field and increased the risk of excessive bleeding, making it difficult to achieve surgical goals such as glansplasty and penile skin closure.

Intraoperative PE is a multifactorial phenomenon, often linked to the anesthetic techniques and drugs used during surgery [4]. The mechanism involves a complex interplay between the autonomic nervous system and local physiological responses. Generally, excitatory parasympathetic outflow (S2-S4) triggered by psychic or local sensory stimulation leads to engorgement of the corpora cavernosa, while inhibitory sympathetic stimulation (T10-L2) induces detumescence by promoting vasoconstriction, opening emissary veins, and enhancing blood drainage.

PE under spinal and epidural anesthesia is believed to result from both psychogenic and reflexogenic stimulation, with a predominance of reflexogenic mechanisms, particularly when the sympathetic blockade extends above the mid-thoracic level [1]. Reflexogenic stimuli arise from instrumentation stimulation of the pudendal nerve (S2,3,4) or in response to tactile stimuli, such as skin preparation before the onset of complete sensory blockade. Another possible explanation is incomplete blockade of the sacral segments of the spinal cord during spinal anesthesia, as the local anesthetic is diluted by the cerebrospinal fluid and its concentration is minimal in areas more distal to the injection site [1]. The erection may also be psychogenic, resulting from exaggerated auditory sensation during the second stage of anesthesia [5]. Despite achieving an adequate sensory blockade up to T8 with 0.5% hyperbaric bupivacaine and fentanyl, our patient experienced a persistent PE, possibly due to incomplete blockade of sacral segments.

PE during general anesthesia is believed to stem from both psychological and reflex-mediated factors. Psychological stimuli, such as sensory input or dreams experienced under anesthesia, may trigger erection. Reflex mechanisms also play a role, with activation of sacral nerve roots due to surgical manipulation or instrumentation of the genital area leading to erection. The suppression of sympathetic vasoconstriction and the alteration of penile vascular resistance by volatile anesthetics contribute to vascular engorgement and PE. Additionally, certain anesthetic agents (like fentanyl and propofol) and sensory input during anesthesia can trigger erections. For the management of PE following spinal anesthesia in our case, intravenous glycopyrrolate and ketamine were administered; however, these interventions failed to resolve the condition. Subsequently, general anesthesia with sevoflurane and atracurium was initiated, aiming to achieve adequate anesthetic depth and complete muscle relaxation. Despite these measures, full detumescence was not attained, necessitating additional therapeutic strategies. Beyond the autonomic nervous system, a complex interplay of non-cholinergic, non-adrenergic, and local neurotransmitters also plays a significant role in this phenomenon [6,7,8].

The management of intraoperative priapism has been addressed in the literature through various methods, including pharmacological approaches such as parasympatholytics and sympathomimetics, which have demonstrated efficacy, albeit primarily documented in case reports. Mechanical interventions, including sustained perineal compression, application of ice packs, and intracorporeal blood aspiration, have also been utilized. Commonly cited pharmacologic treatments for PE under anesthesia involve the administration of intravenous agents such as glycopyrrolate, ketamine, dexmedetomidine, terbutaline, and salbutamol, along with increasing anesthetic depth using inhalational agents [2,3,9]. Additional techniques include dorsal nerve block, intracavernous injection of agonists (phenylephrine, adrenaline), as well as radiological (selective transcatheter embolization therapy) and surgical (arterial ligation or shunts) [6,10]. After spinal anesthesia, various methods were attempted to treat PE, including intravenous glycopyrrolate, ketamine, deepening anesthesia with inhalational agents and muscle relaxants, dorsal penile block, cold compression, and aspiration of intracavernous blood. However, these measures were unsuccessful, leading to prolonged surgery. The patient’s resistant priapism ultimately responded to an intracavernous injection of phenylephrine.

The American Urological Association recommends intracavernous injection of phenylephrine, a selective α-1 adrenergic agonist, for managing priapism, suggesting doses of 100-500 µg for adults, with "lower concentrations in smaller volumes" for pediatric patients, although specific pediatric dosages are not provided [11]. The mechanism is thought to involve α-adrenergic-mediated contraction of cavernous smooth muscle and vasoconstriction of cavernosal arteries. While side effects are rare, they may include hypertension, reflex bradycardia, tachycardia, arrhythmias, headache, and dizziness. Injections should cease once detumescence is achieved.

## Conclusions

PE under anesthesia during pediatric hypospadias repair is a rare but problematic event that can delay and complicate the planned surgery. Various therapeutic approaches have been documented, demonstrating variable efficacy and associated risks. The intracorporeal injection of phenylephrine is a safe, simple, and highly effective method for immediate relief of intraoperative PE in pediatric patients. Given its rapid efficacy and safety profile, it should be considered early in the treatment options for managing PE, whether under regional or general anesthesia.
